# Attitudes and Barriers to Exercise in Adults with Type 1 Diabetes (T1DM) and How Best to Address Them: A Qualitative Study

**DOI:** 10.1371/journal.pone.0108019

**Published:** 2014-09-19

**Authors:** Nadia Lascar, Amy Kennedy, Beverley Hancock, David Jenkins, Robert C. Andrews, Sheila Greenfield, Parth Narendran

**Affiliations:** 1 School of Clinical and Experimental Medicine, University of Birmingham, Edgbaston, Birmingham, United Kingdom; 2 Department of Diabetes, Nuffield House, The Queen Elizabeth Hospital, Edgbaston, Birmingham, United Kingdom; 3 School of Clinical Sciences, University of Bristol, Bristol, United Kingdom; 4 Primary Care Clinical Sciences, University of Birmingham, Edgbaston, Birmingham, United Kingdom; UCL, United Kingdom

## Abstract

**Background:**

Regular physical activity has recognised health benefits for people with T1DM. However a significant proportion of them do not undertake the recommended levels of activity. Whilst questionnaire-based studies have examined barriers to exercise in people with T1DM, a formal qualitative analysis of these barriers has not been undertaken. Our aims were to explore attitudes, barriers and facilitators to exercise in patients with T1DM.

**Methodology:**

A purposeful sample of long standing T1DM patients were invited to participate in this qualitative study. Twenty-six adults were interviewed using a semi-structured interview schedule to determine their level of exercise and barriers to initiation and maintenance of an exercise programme.

**Principal findings:**

Six main barriers to exercise were identified: lack of time and work related factors; access to facilities; lack of motivation; embarrassment and body image; weather; and diabetes specific barriers (low levels of knowledge about managing diabetes and its complications around exercise). Four motivators to exercise were identified: physical benefits from exercise; improvements in body image; enjoyment and the social interaction of exercising at gym or in groups. Three facilitators to exercise were identified: free or reduced admission to gyms and pools, help with time management, and advice and encouragement around managing diabetes for exercise.

**Significance:**

Many of the barriers to exercise in people with T1DM are shared with the non-diabetic population. The primary difference is the requirement for education about the effect of exercise on diabetes control and its complications. There was a preference for support to be given on a one to one basis rather than in a group environment. This suggests that with the addition of the above educational requirements, one to one techniques that have been successful in increasing activity in patients with other chronic disease and the general public should be successful in increasing activity in patients with T1DM.

## Introduction

Type 1 diabetes is a chronic autoimmune disease characterized by the loss of insulin producing cells in the pancreas. It is treated by strict management of lipids, blood pressure and glucose levels, the latter through the injection of insulin [1]. Regular physical activity is also recognized to play a key role in the management of patients with T1DM. It improves insulin sensitivity, reduces cardiovascular risk factors, improves quality of life and reduces mortality [2]. Guidelines for patients with T1DM currently recommend they undertake at least 150 minutes (mins) per week of moderate to vigorous aerobic exercise, spread out during at least 3 days during the week, with no more than two consecutive days between bouts of aerobic activity. They should also be encouraged to perform resistance exercise ‘at least twice weekly on non-consecutive days’ [3], [4].

A large percentage of patients with T1DM do not reach these guidelines [5]. In the Finnish Diabetic Neuropathy Study, 23% of patients with T1DM were classed as sedentary and a further 21% were doing less than 1 session of exercise per week [6].

In a retrospective analysis of the Diabetes and Complications Trial, Makura and colleagues showed that 271 (19%) of the 1441 participants were not achieving the ADA recommendation for activity levels [7]. In a prospective cohort study of 2185 patients with T1DM from 16 European countries (The EURODIAB Prospective Complications Study), Tielemans and colleagues demonstrated that 786 (36%) of patients were doing none or only mild physical activity [8]. To date, there has only been one study that examines barriers to exercise in T1DM. Based in North America and using a questionnaire, this study found that fear of hypoglycaemia, work schedule and loss of control over their diabetes were the main barriers to exercise [9]. They also identified that knowledge of insulin pharmacokinetics, awareness of strategies to reduce hypoglycaemia, and increased social support (including having someone to exercise with) was associated with fewer barriers. Culture, diet and diabetes education vary significantly between countries [10] and therefore these findings in the North American population urgently need to be validated in other populations. By interviewing adults with T1DM this study aimed to qualitatively explore attitudes and barriers to the uptake and maintenance of exercise. This should provide a more in-depth understanding than that provided by questionnaires, and should allow the development of strategies to increase exercise to the recommended levels in patients with T1DM.

## Methods

### Sampling and recruitment

Purposeful sampling to include both genders and across all adult age groups was used to recruit adults with a clinical diagnosis of T1DM to participate in individual face to face interviews.

Patients aged between 18 to 80 years were recruited from the Diabetes Unit of Selly Oak Hospital, University Hospitals Birmingham NHS Foundation Trust, Birmingham, UK. Patients were approached to participate in the study whilst waiting for their diabetes appointment, and/or directly by their clinician at the end of their diabetes appointment. If patients were interested in taking part in the study, their details were taken and information sheets were sent to them. They were then contacted to confirm they remained interested in taking part in the interview, and a date scheduled for interview.

### Ethics statement

Ethical approval was granted by Birmingham East North and Solihull Research Ethics Committee. The study was sponsored by The University of Birmingham, UK. Informed written consent was obtained from all the participants. All participants were adults older than 18 years.

### Interviews

Each interview lasted an average of 30 minutes and was undertaken in the Diabetes Unit of Selly Oak Hospital. We ensured that the clinicians and allied health care professionals who were involved in managing the patient were not involved in any way in the interview so that the patient responses would not be influenced by their presence.

All interviews were carried out by one experienced qualitative researcher using a topic guide (see Table 1) which asked about patients' level of exercise, reasons why they do not exercise, and barriers to initiation and maintenance of an exercise programme. Resistance to change, individual needs and preferences, use of exercise diaries, approaches to monitoring levels of exercise and acceptability of different types of intervention delivery were also explored. At the beginning of the interview patients were asked about their diagnosis, family history of T1DM, treatment regime and control, to provide the context to explore each individual's relationship with diabetes and how this might impact on their activity and exercise behaviour. All patients were made aware that the interview would be recorded, transcribed and analysed anonymously.

**Table 1:** Interview schedule.

1	Can you tell me a little bit about your diabetic history: when were you first diagnosed, has anyone else in your family had diabetes, how your diabetes is managed, that sort of thing?
2	How does the diabetes affect your lifestyle?
3	As you know, the research is about physical activity. What kinds of physical activity do you do?
4	Do you do any exercise or take part in any sports?
5	Would you like to be more physically active?/Would you like to do more exercise?
6	What do you think prevents you from taking exercise?
7	What would help or encourage you to do more exercise?
8	Some things have been suggested that might help people to take up, and keep doing, more exercise. Would any of these be of interest to you?- one to one advice from a health and fitness advisor- attending an exercise group organised by the hospital or your GP- motivational support – someone who keeps in touch to see how you are doing with your exercise programme
9	The latest health recommendation is that we all undertake a minimum of 150 minutes of exercise a week – half an hour, five times a week. What do you think about that? Do you think you could achieve that target?
10	Follow up: what is the one thing that would most help you to increase your level of exercise?

### Data handling and analysis

The interviews were recorded and transcribed. Data were analysed using a framework approach [11] which involved reading through the transcripts and developing a matrix of overarching and supporting themes.

## Results

### Patients

Over a four month period (April to July 2010), 48 patients expressed interest in the study. Six did not respond to repeated telephone messages and five were unavailable for interview because of their work or living away. Interviews were arranged with 32/48 patients and six subsequently failed to attend or cancelled at very late notice.

A further five, recruited towards the end of the study, were not interviewed because they belonged to subgroups already well represented in the sample and also because data saturation had been achieved. In total 26 interviews were undertaken.

### Characteristics of the sample

12 females and 14 males were interviewed (Table 2). The females were aged between 21 and 62 years (mean 40.7±13.9 years) and the males were aged between 21 and 65 (mean 42.6±13.3). Time since diagnosis ranged from two weeks to 50 years (mean 17.6±15.7 years), and age at diagnosis ranged from nine months to 57 years (mean 25.3±14.1 years). The treatment regime was insulin injections for 19 patients. Five patients used an insulin pump. The treatment regime was not recorded for two patients.

**Table 2:** Interviewee Characteristics.

Interview order	Gender	Age	Age diagnosed	Time since diagnosis	Treatment
1	M	57	49 years	7 years	Injection
2	M	21	20 years	10 months	Injection
3	F	23	18 years	5 years	Injection
4	M	31	29 years	Less than 2 years	Injection
5	F	54	27 years	27 years	Pump
6	F	21	9 years	12 years	Pump
7	M	34	32 years	18 months	Injection
8	F	38	31 years	7 years	Injection
9	M	33	33 years	2 weeks	Injection
10	M	55	43 years	12 years	Injection
11	F	24	9 years	15 years	Pump
12	M	53	27 years	26 years	Injection
13	M	64	14 years	50 years	Injection
14	F	40	25 years	15 years	Pump
15	F	39	21 years	18 years	Injection
16	F	33	21 years	12 years	Injection
17	F	50	10 years	40 years	Injection
18	M	35	30 years	5 years	Injection
19	M	50	45 years	5 years	Injection
20	M	50	9 months	50 years	Injections
21	F	62	19 years	43 years	Pump
22	M	44	28 years	16 years	Injection
23	M	22	13 years	9 years	Not recorded
24	M	47	4 years	43 years	Not recorded
25	F	59	57 years	Nearly 3 years	Injection
26	F	45	42 years	3 years	Injection

Framework analysis of the data revealed three major themes and supporting themes around exercise: physical activity and exercise behaviour; barriers to exercise; facilitating and encouraging exercise (see Table 3).

**Table 3:** Interview Themes.

Overarching themes	Supporting themes	
Activity and exercise behaviour	Physical activity	
	Exercise behaviour	
	Perceptions of activity and exercise levels	
	Present and desired activity levels	
	Achieving targets	
Barriers to exercise	Time, work and lifestyle	
	Health and medical	
	Social and personal	
	Environmental	
Facilitating, motivating and encouraging exercise	**Facilitators and Motivators**	**Interventions to encourage exercise**
	Enjoyment	One to one advice from a health and fitness advisor
	Rewards	Attending an exercise group organised by the hospital or GP
	Support and encouragement	Motivational support
	Access	
	Knowledge and advice	
	Time management	

### Theme 1: Physical activity and Exercise Behaviour

#### Activity levels

Patients were asked to describe how active they were in terms of lifestyle and work.

A recurring theme was interviewees confusing an *active* lifestyle with a *busy* lifestyle.

Many cited walking when describing activity levels but the amount of walking varied and few walked a total of thirty minutes or more on a daily basis. Having a dog to walk or not having a car was associated with regular walking but people with cars were more likely to drive than walk, often citing the distances they had to cover between home and work as being the main reason although bad weather and not having time to walk were additional reasons. A number of participants described walking for pleasure but probing revealed that this usually meant when they were on holiday and not as a regular activity. Similarly, six people (all men) said they either cycled occasionally or that they had been cyclists when they were younger, though they were not currently regular cyclists.

#### Exercise behaviour

Over half of the group (16) claimed to do some form of exercise but probing revealed that more than half of them only exercised an average of once or twice a week and sometimes not at all; some described “going through phases”.

Swimming and going to the gym were the most frequent forms of exercise. Individuals participated in a range of other exercise activities and sports including fencing, badminton, tennis, yoga and skiing but not on a regular (weekly) basis. Although some of the men had previously played football when they were younger, none of the participants took part in team sports currently.

Whilst swimming was given as an example of preferred exercise by half of the people (13), it emerged that half of them (7) only swam occasionally such as when they were on holiday. Three people explained that although they enjoyed swimming they were worried about doing so because they felt less in tune with their bodies when they were in the water and at greater risk of a hypoglycaemic attack. Two people did not swim because they were embarrassed at being seen in swimwear.

One third of participants (9) stated that they exercised at a gym and three of these went to a gym once a week or less. Although a couple of the regular gym users identified social as well as health reasons for going, most gave the impression that they had to make an effort to go but did so for the health benefits they derived.

Some of those who did not use a gym volunteered why: they believed they could exercise equally well elsewhere, they preferred to take part in a sport or activity outside or they felt they weren't “gym people”.

#### Increasing activity levels

Participants were informed of the health recommendation that they take a minimum of 150 mins of moderate intensity exercise every week. A third (9) claimed that they were achieving that target (when they included walking as part of their regime). Many of these people did not exercise for 30 mins a day on five days a week, but rather longer bouts of exercise on fewer than five times a week.

Of those 17 people that said they were not achieving the target, five thought it was possible for them to increase their exercise levels to achieve the target. Seven people did not believe they could achieve the target, and some suggested that the target was unrealistic.

Well over half (18) said they would like to be more active. The reasons why they did not exercise and the things that might help them to be more active are discussed in the next two themes.

### Theme 2: Barriers to Exercise

Participants identified four main barriers ‘health and medical’, ‘time’, ‘work and lifestyle’, ‘social and personal’ and ‘environmental’, to exercise.

#### Health and Medical

Four people cited non-diabetes related health problems as reasons for not exercising: asthma, arthritis and sports related injury. More people (nine) attributed their under active lifestyle to diabetes, although it was not necessarily a barrier for them. Many more than this talked about exercise and diabetes in ways that illustrated that they recognised a link between exercise and management of diabetes but many did not fully understand the relationship and how blood sugar could be affected by activity.

*“I'm never sure why my sugar levels do actually shoot after I've exercised. They can go up really high and sometimes that is not a worry but it's just like why can't I suss this? I still don't understand what the logic is behind that.”*

(Int 15, F, 39)

Many participants talked about the risk of hypoglycaemia when exercising but very few cited fear of a hypoglycaemic attack as a specific reason for not exercising.

Similarly, complications due to diabetes were another potential condition-related barrier to exercise but it tended to influence the type of exercise or limit the amount of exercise people could do rather than stop them completely.

#### Time, work and lifestyle

People's lifestyles, their work and demands on their time was a far greater barrier than diabetes for most people. Half (12) cited lack of time as a barrier. Working full time, the type of work people did and shift work were all described as restricting people's ability to exercise. Added to this were demands in the home or caring for children or relatives.

However, several interviewees admitted that the problem was not so much lack of time but more a problem of time management or prioritisation. However, when combined with other factors – such as low motivation to exercise, bad weather, erratic lifestyle – a perceived lack of time became the excuse for not exercising.

#### Social and personal

A wide range of additional factors contributed to individual reluctance to exercise. None of them were major factors for the group but they influenced individual behaviour.

Lack of motivation to exercise was described by four people, another three more bluntly stated that they were lazy and among those who described time as a barrier it emerged that not prioritising exercise was at least partly due to a lack of interest in being more active.

*“I don't know that if I'd not been a diabetic I'd be doing any more exercise than in fact I am. My interests tend to be more mental, cerebral than physical anyway and I don't think the fact I was diagnosed diabetic has really had much impact on that. Not to my knowledge. That's how I feel about it, anyway.”*

(Int 24, M, 47)

Six participants felt inhibited to exercise because of embarrassment or fear of failure. This was not restricted to a specific gender or age group. Three participants related embarrassment to body image but it was also related to fears of not being as good as other people.

#### Environmental

Access to a gym or swimming pool was raised as a problem by a few people, usually in relation to finance although several interviewees pointed out that there were a number of local authority initiatives in the area that provided free or reduced cost access to facilities. However, the concession often applied during the day, not at evenings or weekends when some of the employed people or students would prefer to use the facility.

Another issue was weather. Four people specifically stated that bad weather – or even good weather if the temperature was too high – stopped them from cycling or walking.

### Theme 3: Facilitating and encouraging exercise

Participants were asked about what would help or encourage them to take more exercise and we called these Facilitators (free or reduced admission to gyms and pools) and Motivators (health benefits, body image, enjoyment, support and encouragement, advice and information, time management).

Although only a small number of people cited cost as a barrier to exercise, more thought that free passes or reduced membership of gyms and swimming pools would be a facilitator. Interestingly some of them were aware of council schemes providing this, yet they did not take advantage of the schemes. The social aspect of exercise was seen as helping with motivation. Several interviewees, especially older people (over 50) with partners, explained that they liked to exercise with someone else. Those with children also expressed a preference for physical activities that they could do as a family. These groups usually referred doing more walking or swimming to increase their activity levels.

The Social Facilitator also included a practical, security element, e.g. when applied to swimming or going to a gym so that someone could spot if they were having a hypo.

Some interviewees felt that the health benefits were an important motivator to exercise:

*“I'd do anything to help my body now if it means that the diabetes is going to live healthier if that makes sense, if I've going to manage it better, then I'll do anything.”*

(Int 9, M, 33)

Investing in their health avoiding complications in the future and improving body image were all quoted as motivators.

Enjoyment seemed to be an important element of exercise: the interviews contained many examples of how people enjoyed a wide range of activities. Meeting the target of 150 mins of exercise each week was more common among those who described the pleasure they derived from exercise. Consequently, finding an exercise or a sport that people enjoyed emerged as an important factor in motivating people to exercise.

Among the barriers to exercise were lack of motivation and a perceived lack of time so providing external motivators was important for some people. This took various forms but convenience and access to people who could help them were important elements.

A quarter of interviewees said that advice or information about what exercises and why would encourage them to exercise more. This would help them to understand the relationship between exercise and blood sugar levels.

Allied to this was the power of medical advice to influence behaviour:

*“If they said to me, ‘Look, you’ll extend your life if you do more exercise,’ obviously you would. Or, ‘You’d feel better in yourself.' Obviously you'd try your damned hardest to do more exercise…If he said it genuinely. If he said, ‘Look, I'm serious.’*

(Int 1, M, 57)

Time has already been identified as a perceived barrier to exercise and consequently, having more time was posed as the solution.

While creating more time is clearly not possible, time management can release time and opportunity for exercise, a conclusion that some participants came to as the interview progressed.

### Encouraging exercise

Interviewees were presented with three interventions aimed at encouraging exercise and asked for their views on each.

#### Option 1: one to one advice from a health and fitness advisor

This was the intervention that appealed to the greatest number of people (20 people). There were three main reasons given for supporting this intervention. The first was the perception that one to one advice would be tailored to individual needs and provided the opportunity to learn how specific exercises or gym equipment could be used to work to personal goals such as improving fitness or losing weight.

Seven participants stressed that the personal trainer should be someone who understood diabetes and was considered helpful because it provided an external motivator.

#### Option 2: attending an exercise group organised by the hospital or General Practitioner (GP)

Half of the participants (13) supported the idea of an exercise group organised by the hospital or GP. The main attraction was the social aspect of being in a group and having peer support that comes with being part of a group.

The composition of the group was raised as an important factor, reinforcing the desirability of being with “likeminded people” (Int 20, M, 50) although this meant different things for different people: age, similar level of fitness, other diabetics or a mixed group including non diabetics.

*“I'd like it to be people my age”*

(Int 14, F, 40)

*“That would be a brilliant idea. Brilliant, yeah. I would go to it if I knew that it was run by someone that understands it and that there'd be other diabetics there. I would go, definitely.”*

(Int 26, F, 45)

The main objections to the group intervention were almost the opposite of the reasons for supporting the idea: a dislike of group situations and preconceived ideas of who would be in the group and not wanting to identify with them.

*“I don't really like group activities. I'd much rather, when I exercise, do things by myself or I go with a member of the family to play a sport. I'm not so keen on the group activities.”*

(Int 6, F, 21)

There were further reasons for not supporting this type of intervention. There was a degree of embarrassment among some of those who said they disliked group exercise. Several interviewees did not think they could commit to a regular group and others felt they did not need the intervention because they didn't need to increase their activity levels.

#### Option 3: motivational support

Eleven people supported the third intervention, motivational support, but it was the least popular of the three options and 12 actively expressed a dislike. A third of interviewees (8) thought it might help to motivate them and encourage them to meet their goals.

The main reasons for rejecting option 3 were that it would be easy to deceive the person providing support and the perception that this approach would be “childish” or “nagging”.

*“That would be a no no. I wouldn't mind going to like your Weightwatchers, things like that because you're choosing to go there. And again that guilt thing, I've got a terrible guilt thing where food and exercise are concerned. And knowing that someone was going to phone me to check up, no, I wouldn't like that.”*

(Int 8, F, 38)

One interviewee aptly summarised the potential benefit of support from a mentor but not by telephone:

*“It would be great if it was over the phone because you could lie madly. 'Oh yes, I've lost two stone and I'm really trim. ‘I’m a great believer in mentors; I really am. But I think the mentor has to be tangible. You have to be able to go and see them and you have to care about whether they approve or encourage you or not. But no, not a voice over the phone.”*

(Int 25, F, 59)

## Discussion

### Statement of, and reflection on the principal findings

Six main barriers to exercise were identified in this study (see Table 4). These were: lack of time; work related factors; difficulties with access to facilities (distance and cost); lack of motivation; low confidence (embarrassment about body image and fear of failing); weather; and health specific barriers (low levels of knowledge about managing diabetes around exercise and diabetes complications).

**Table 4:** Barriers, Facilitators and Motivators.

BARRIERS TO EXERCISE
Lack of knowledge on the management diabetes for exercise
Time and work
Access to facilities (distance and cost)
Embarrassment, body image, fear of failure
Lack of motivation
Weather

MOTIVATORS	FACILITATORS
Health benefits	Free or reduced admission gyms/pools
Body image	Better time management
Enjoyment	Support and encouragement
Social Aspects	Advice and information

The first five of these barriers have been reported in many studies of barriers to exercise: individuals at high risk [12] or already affected by type 2 diabetes [13]–[17]; people with disabilities and chronic health conditions [18]–[20]; people with cancer [21]; and the general population [22], [23] have identified these factors as barriers to exercise.

Only one study has looked at barriers to exercise in patients with T1DM. Using a questionnaire, Brazeau et al, identified four main barriers to exercise in 100 patients with T1DM: fear of hypoglycaemia; work schedule; loss of control over diabetes; and low fitness level.

Our study identified similar barriers to exercise. Many patients stated that work schedules were a barrier to exercise. Whilst many patients were aware of the risks of hypoglycaemia with exercise, the fear of hypoglycaemia was not specifically stated as a reason for not exercising. However a few patients did allude to this fear, mentioning that one advantages for going to the gym or swimming pool was that they would be around people who would notice if they went hypo. Again although patients did not explicitly say that loss of diabetes control was a barrier to exercise, many patients expressed frustration at not understanding why in certain situations their blood sugars rose with exercise, and felt that having more information around how exercise can affect glucose levels in diabetes would encourage them to exercise. In addition we found that the presence of diabetes related complications and other co-morbidities was not a major barrier to exercise in this study, though it did influence the type of exercise that patients chose to undertake.

Our interviews identified 4 main factors that motivated patients to exercise: physical benefits from exercise; improvements in body image; enjoyment of the exercise and the social interaction of exercising at gym or in groups.

These factors have also been found to be important in individuals at high risk [12] or already affected by type 2 diabetes [13]–[17]; people with disabilities and chronic health conditions [18]–[20]; people with cancer [21]; and the general population [22], [23]. Weight control, which was cited as an incentive to exercise by people with pre-diabetes, type 2 diabetes [14], [17] and cancer [21], and positive emotions and improvements in mental well-being, cited by cancer patients [21], were not cited as motivators to exercise in our patients.

Three factors were identified by our patients that would encourage them to increase their exercise levels. These were free or reduced admission to gyms and pools, help with time management, and better advice about what exercise they should do, why and how this would affect their blood sugars. Similar findings have been found in other populations [24].

In order to overcome exercise barriers, other studies have found community-based exercise programmes [23], [25] and group activities [20] to be the most useful programmes with people with disabilities or chronic health conditions stating that these programmes were helpful as they could exercise with patients of similar abilities and share their experiences of their illnesses [21]. In contrast our T1DM patients preferred individually tailored exercise support, designed with consideration of their preferences, circumstances and fitness levels. They also were not keen on motivational interviewing, particularly if telephone based, a well accepted approach to promote exercise among people with other health problems [20], [26], [27].

### Strengths and weaknesses of the study

The strengths of this study are that it is the first qualitative study of barriers and facilitators of exercise for patients with T1DM. It includes male and females with a broad age range (21–64) and diabetes duration, and on both insulin injection and pump therapy. The weaknesses are that patients were only recruited from one clinical centre, ethnic diversity was low and the majority were inactive.

### Conclusions and recommendations

In this study we have shown that many of the barriers, motivators and facilitators of exercise in patients with T1DM are similar to patients with other chronic diseases and the general public. The only difference are that patients with T1DM require education about the effect of exercise on diabetes control and they prefer support to be given on a one to one basis rather than in a group environment. This suggests that with the addition of the above educational requirements, one to one techniques that have been successful in increasing activity in patients with other chronic diseases and the general public should be successful in increasing activity in patients with T1DM.
